# An investigation of the effect of nurses’ technology readiness on the acceptance of mobile electronic medical record systems

**DOI:** 10.1186/1472-6947-13-88

**Published:** 2013-08-12

**Authors:** Kuang-Ming Kuo, Chung-Feng Liu, Chen-Chung Ma

**Affiliations:** 1 Department of Healthcare Administration, I-Shou University, No.8, Yida Rd. Yanchao District, Kaohsiung City 82445, Taiwan, R.O.C; 2 Department of Information Management, Chia-Nan University of Pharmacy and Science, No.60, Erh-Jen Rd., Sec.1, Jen-Te District, Tainan City 71710, Taiwan, R.O.C

**Keywords:** Mobile electronic medical record (MEMR), Nurses, Technology readiness index (TRI), Technology acceptance model (TAM)

## Abstract

**Background:**

Adopting mobile electronic medical record (MEMR) systems is expected to be one of the superior approaches for improving nurses’ bedside and point of care services. However, nurses may use the functions for far fewer tasks than the MEMR supports. This may depend on their technological personality associated to MEMR acceptance. The purpose of this study is to investigate nurses’ personality traits in regard to technology readiness toward MEMR acceptance.

**Methods:**

The study used a self-administered questionnaire to collect 665 valid responses from a large hospital in Taiwan. Structural Equation modeling was utilized to analyze the collected data.

**Results:**

Of the four personality traits of the technology readiness, the results posit that nurses are optimistic, innovative, secure but uncomfortable about technology. Furthermore, these four personality traits were all proven to have a significant impact on the perceived ease of use of MEMR while the perceived usefulness of MEMR was significantly influenced by the optimism trait only. The results also confirmed the relationships between the perceived components of ease of use, usefulness, and behavioral intention in the Technology Acceptance Model toward MEMR usage.

**Conclusions:**

Continuous educational programs can be provided for nurses to enhance their information technology literacy, minimizing their stress and discomfort about information technology. Further, hospital should recruit, either internally or externally, more optimistic nurses as champions of MEMR by leveraging the instrument proposed in this study. Besides, nurses’ requirements must be fully understood during the development of MEMR to ensure that MEMR can meet the real needs of nurses. The friendliness of user interfaces of MEMR and the compatibility of nurses’ work practices as these will also greatly enhance nurses’ willingness to use MEMR. Finally, the effects of technology personality should not be ignored, indicating that hospitals should also include more employees’ characteristics beyond socio-demographic profiles in their personnel databases.

## Background

For decades, governments and healthcare reform advocacy groups have urged that healthcare information technologies (HITs), such as electronic medical record (EMR) and computerized physician order entry (CPOE) systems, should be utilized extensively as a means to enhance patient safety, improve healthcare service quality, and reduce costs [1]. More specifically, due to the advances of technologies in mobility and ubiquity, the adoption of mobile HITs, such as mobile EMR (MEMR), is growing rapidly. Mobile HITs are believed to be of significant benefits in improving point-of-care [2].

The adoption of MEMR can help nurses accomplishing their daily care routines and record-keeping jobs more efficiently and effectively. However, MEMR requires heavy capital investment and sophisticated arrangement of wireless network architectures. Further, HITs cannot improve organization’s performance if they are not utilized [3], but HITs are often seriously hampered by healthcare staff who is expected to benefit from its use [4]. Thus, to maximize MEMR’s benefits, it is critical for hospitals to obtain an in-depth understanding of the motives that affect nurses’ intentions toward using MEMR.

Research issues such as the adoption behavior of innovative IT and the diffusion of innovations have attracted attention in literature for decades [5] and has proven to be one critical issue for researchers [3]. Traditionally, technology adoption has been investigated via constructs such as usefulness and ease of use for predicting individual’s acceptance of technologies [6]. However, such a viewpoint may not afford understanding and clarification the determinants of ease of use and usefulness perceptions when people accept a technology [7]. Hence, more in-depth studies might be required to explore this issue to propose better interventions to improve people’s acceptance and use of a technology.

Of the various antecedents, individual’s internal differences (i.e., personality traits) are often investigated [4,8]. If the influence of individual’s personality traits on the adoption of a technology is ignored, then there is a risk that adoption models may be mis-specified and, in turn, influence people’s adoption of a technology negatively. Among the differing personality traits, Parasuraman [9] asserted that the Technology Readiness Index (TRI) is conceptualized as a trait, which is a relatively stable descriptor of individuals.

Thus, the aim of this study is to explore how nurses’ technology readiness and perceptions of MEMR influence their intentions toward MEMR use. This study takes individual differences into account by integrating the construct of individuals’ technology readiness [9] with the Technology Acceptance Model (TAM) [6] may contribute to the knowledge of innovative adoption research.

### Technology readiness index

Technology readiness (TR) refers to “*people’s propensity to embrace and use new technologies in order to accomplish goals in their home life and at work*” [9]. The construct can be regarded as an overall state of mind originating from both mental drivers and inhibitors that jointly determine a person’s tendency to use new technologies [10].

Parasuraman [9] proposed the TRI can be used with general consumer populations. It is comprised of 36 items belonging to four dimensions: (1) optimism or “a positive view of technology and a belief that it offers people increased control, efficiency, and flexibility in their lives”; (2) innovativeness or “a tendency to be an early adopter of technology and opinion leader”; (3) discomfort or “a perception of unable to control the technology and a feeling of being overwhelmed by it”; (4) insecurity or “suspect of technology and doubt about its capability to work”. Among the four dimensions, optimism and innovativeness are the enablers of technology readiness, whereas discomfort and insecurity are inhibitors [9]. According to Parasuraman’s work [9], people who possess optimism and innovativeness and less discomfort and insecurity are more prone to accept and use a new technology.

Previous literature of TRI can be roughly classified into two categories. The first type of study assesses the subjects’ technology readiness scores by leveraging TRI [11-14]. Most results reveal that the TRI scores are different among different subjects in different context. The second type of study uses TRI with other theory to investigate individuals’ acceptance of new technologies [5,8,10,15-17].

Despite that most studies support that TRI can predict the acceptance of new technologies, however, none of these studies are empirically validated in healthcare context which is rather more complex than other industries [18]. Further, the results of previous literature may also not be applicable in healthcare setting. Moreover, Liljander and colleague [5] argued that more studies are still required to further investigate the ability of TR in predicting people’s acceptance of technology. Since TRI is currently the most integrative measure of technology readiness [17], it is both theoretically appropriate and comprehensive to investigate nurses’ behavioral intentions of using MEMR.

### Technology acceptance model

Based on the causal relationship of the Theory of Reasoned Action proposed by Fishbein and Ajzen [19], Davis [6] proposed the TAM that identified perceived usefulness (PU) and perceived ease of use (PEOU) as the two salient beliefs that determine people’s attitude toward accepting a technology. Davis [6] also concluded that PU has a stronger relationship with user acceptance of a technology than PEOU does. TAM has been applied in various contexts and has received empirical support from numerous studies [20].

Despite the vast support for TAM, researchers have called for others to explore whether TAM’s belief variables (i.e., PU and PEOU) are mediators of the effect of antecedent variables [21]. In healthcare setting, previous studies have adopted TAM to investigate the acceptance of differing information systems on hospital staff, including physicians [22-28], nurses [29-33], specific healthcare professionals [34-40] (e.g., occupational therapists, physiotherapist, etc.), or mixed groups [41-44]. Further, individuals including patients are also frequently investigated [45-50]. Regarding the antecedents of PU/PEOU constructs, many of these studies did not explore such influencing factors [22,25,27,30,32,39,41,51]. Other studies in healthcare context that investigated the antecedents of PU and PEOU are still scarce [28,35,38,43,49], which might limit our understanding of this issue. More endeavors are thus required to probe on this issue. Consequently, this study employed personality traits (i.e., technology readiness) as antecedents of PU and PEOU to further advance our knowledge on this issue and to fulfill the calling of previous literature [21]. Table 1 reveals a review of TAM studies in healthcare setting.

**Table 1:** TAM studies in healthcare

**Studies**	**Subjects**	**Technology studied**	**Antecedents of PU/PEOU**
Xue et al. [49]	Ageing women	Mobile health informatics	Perceived user resource, technology anxiety*, perceived physical condition
Pynoo et al. [27]	Physicians	Picture Archiving and Communication Systems (PACS)	None
Hung et al. [25]	Physicians	Medline system	None
Holden et al. [30]	Nurses	Bar coded medication administration technology	None
Aldosari [41]	Healthcare professionals	PACS	None
Or et al. [47]	Patients	Web-based self-management system	None
Lim et al. [46]	Females	Seeking health information via mobile phone	None
Kowitlawakul [31]	Nurses	Telemedicine	Years working in hospital, support from physicians, support from administrators
Yu et al. [40]	Healthcare professionals	Health IT	Subjective norm, image, age, job level, work experience, computer skills
Xue et al. [48]	Females	Female-focused healthcare applications	Output quality, result demonstrability, subjective norm, image
Aggelidis and Chatzoglou [43]	Healthcare Professionals	Health IT	Social influence, training, facilitating conditions, anxiety*, self-efficacy
Tung et al. [33]	Nurses	Electronic logistic IS	Compatibility, trust
Wu et al. [44]	Healthcare professionals	Mobile computing	Compatibility, self-efficacy, technical support and training
Schaper and Pervan [38]	Occupational therapists	ICT	Compatibility, organizational facilitating conditions, computer anxiety*, computer self-efficacy
Kim and Chang [45]	Adults	Health information website	Information search, usage support, customization, purchase and security
Chen et al. [29]	Nurses	Web-based learning	Demographic data (e.g., age, educational level, nursing job experience, job position, previous web-based learning experience, etc.)
Yi et al. [28]	Physicians	Personal Digital Asistant (PDA)	Personal innovativeness in it*, result demonstrability, image, subjective norm
Pare et al. [26]	Physicians	Clinical IS	Psychological ownership
Liu and Ma [37]	Healthcare professionals	Service oriented medical records	Perceived service level
Liu and Ma [36]	Healthcare professionals	e-services for EMRs	Perceived system performance
Wilson and Lankton [50]	Patients	e-health	Intrinsic motivation
Barker et al. [34]	Healthcare professionals	Spoken dialogue system	Product characteristics
Liang et al. [35]	Healthcare professionals	PDA	Compatibility, job relevance, support, personal innovativeness*
Chismar et al. [24]	Pediatricians	Internet-based health applications	Experience, subjective norm, image, job relevance, output quality, result demonstrability
Van Schaik et al. [39]	Physiotherapists	Portable postural assessment system	None
Chau and Hu [23]	Physicians	Telemedicine	Compatibility, peer influence
Chau and Hu [51]	Physicians	Telemedicine	None
Rawstorne et al. [32]	Nurses	Patient care IS	None
Hu et al. [22]	Physicians	Telemedicine	None

Note: * denotes personality traits related constructs, PEOU denotes perceived ease of use, PU denotes perceived usefulness.

To sum up, TAM is a theory that has undergone a number of changes such as TAM2 [21,52], Unified Theory of Acceptance and Use of Technology (UTAUT) [53], or TAM3 [54]. Each theory has contributed profoundly to the acceptance knowledge. Since the main purpose of this study is to investigate the influence of personality traits on the acceptance of MEMR, the parsimonious and powerful TAM [20,21,55] was chosen to segregate the impact of other factors proposed by these TAM-based theories.

### Mobile electronic medical records

A variety of definitions for EMR, or similar terms such as computer-based patient records (CPR) and electronic health records (EHR) have been paralleled. The Institute of Medicine (IOM) published their landmark report, “The Computer-based Patient Record: An Essential Technology for Health Care” which defined CPR as an “*electronic patient record that resides in a system designed to support users through availability of complete and accurate data, practitioner reminders and alerts, clinical decision support systems, links to bodies of medical knowledge, and other aids*” [56]. Indeed, the rapid development of wireless and handheld electronic devices has urged the progress of the “mobilization” and “wireless technology” of EMRs. Many researchers also started to explore mobile EMR-related issues [2,57-61]. However, these studies were mainly focused on the development or applications of MEMR, and less concerned on investigating the factors influencing the adoption of MEMR by nursing staff [44,58]. Referring to Hsu and colleague’s work [58], the present study defines MEMR as “an EMR that can be accessed and managed via wireless or mobile devices to help health professionals acquiring patients’ information anywhere and anytime”.

### Status quo of technology development in Taiwan

According to the latest “Global Information Technology Report 2012” published by the World Economic Forum in 2012 [62], Taiwan ranks 11th for being most innovative and digitized nations in the world. The National Communications Commission (NCC) of Taiwan [63], reported that in 2012, the number of mobile phone accounts of the first three quarters of 2011 amounted to 28,610,000 (an average of 123.3 accounts per hundred people); 71.2% of the accounts have enabled the mobile Internet functions. The Foreseeing Innovative New Digiservices (FIND) [64], an authoritative website that provides abundant and professional information on Internet demographics and trends in Taiwan, predicted that in 2015, the people in Taiwan having smart phones and Tablet PCs will reach the level of 52.5% and 26.4%, respectively. These might imply that people in Taiwan are accustomed to use these most up-to-date and popular technologies in their daily life.

Today, cultivating information skills is one of the basic goals of nursing education (e.g., word processing and spreadsheet software) in Taiwan. Nursing schools usually invite many experts for lectures or providing short courses relating to advanced information management (e.g., exploring the issues of patient privacy and information security). This demonstrates that most nurses should have acceptable information literacy after graduation in Taiwan.

### Research framework and hypothesis development

This study integrates TAM and TRI due to the following reasons. First, both TAM and TRI can be used to explain peoples’ technology acceptance [6,9]. Second, the major difference between these two models lies in that TAM uses system-specific perceptions to explain technology acceptance while TRI is via individuals’ general inclination [17]. Third, individual differences (i.e., psychological traits) are mediated by the cognitive dimensions (i.e., PU and PEOU) in predicting people’s acceptance of technology [65]. Thus, it is theoretically appropriate to integrate TAM with TRI to investigate nurses’ acceptance of MEMR.

**Figure 1:**
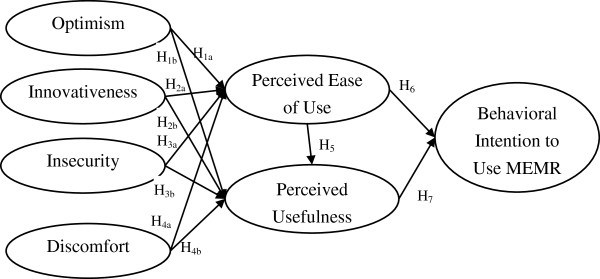
Research framework.

Walczuch and colleague [8] combined the TRI and TAM into one model to measure the relationship between the personality traits from TRI and the cognitive factors from TAM [6]. It was initially used in financial service settings and gave valuable insights in a technology readiness study. However, it did not investigate the relationships between PU, PEOU and behavioral intention, simultaneously, in one model. Further, the healthcare industry has markedly different social and technical context compared with other industries [66]. Thus, Walczuch and colleague’s [8] results may not be fully applied to this study. More in-depth investigations are required. To better understand the abovementioned relationships, this study proposed a research framework, as depicted in Figure 1. The framework involves the basic concept of TAM, which indicates nurses’ intention to use MEMR and will be affected by their PEOU and PU of MEMR. The TRI’s four indicators (optimism, innovativeness, discomfort and insecurity) are regarded as exogenous variables which will influence nurses’ PEOU and PU toward MEMR.

Despite the relationships among constructs proposed in this study had been supported by numerous healthcare studies, these studies employed different subjects, technologies, research methodologies, etc. These differing research designs might have influences on the results, thus we argue that more in-depth studies are required to accumulate and advance our knowledge on technology acceptance in healthcare context. Further, EMRs are assumed to improve healthcare quality plus nurses are the majority staff that might have great impact on healthcare quality. How nurses might accept MEMR will be an important issue to be investigated. Hence, by adding personality traits constructs, gaining a deeper understanding of factors influencing PU and PEOU are highly expected.

#### Effect of optimism on PEOU and PU

Optimism is a general construct that catches peoples’ specific feelings and indicates that technology is a good thing [14]. A technology optimist holds that new technology will tender people more benefits such as increased control, flexibility, and efficiency in their lives [9]. They use more optimistic strategies which are usually more effective in realizing the expected results [8]. In other words, optimists are less likely to focus on negative events and will accept technology more freely. Thus, optimists perceive technologies as being more useful and easy to use in that they are less irritated about the negative outcomes of technology. Two hypotheses are proposed according to previous discussions:

H_1a_: Optimism positively influences nurses’ PEOU of MEMR.

H_1b_: Optimism positively influences nurses’ PU of MEMR.

#### Effect of innovativeness on PEOU and PU

Innovativeness refers to the tendency to be a technology pioneer and thought leader [9] and is regarded as an important determinant of cognitive absorption which, in turn, influences PEOU and PU [67]. This indicates that individuals with high technology innovativeness have stronger intrinsic motivations to accept new technology and even enjoy the sensation of trying new technology [17]. Agarwal and Prasad [68] asserted that personal innovativeness is important when investigating the acceptance of innovative technology. Furthermore, people scoring high in innovativeness have, in general, a positive impression of a new technology’s usefulness [8]. According to previous discussions, this study proposes the following hypotheses:

H_2a_: Innovativeness positively influences nurses’ PEOU of MEMR.

H_2b_: Innovativeness positively influences nurses’ PU of MEMR.

#### Effect of insecurity on PEOU and PU

Insecurity, in the context of this study, is defined as a distrust of technology and skepticism about its ability to work properly [9]. People who are high in insecurity usually short of confidence about the security of new technologies and they often ask for assurance [69]. That is, they may feel that some risks might exist when using new technology. Previous studies [70,71] confirmed that risk perceptions influence PU and PEOU. It is only when people believe that they will acquire great advantage from using the new technology that they are willing to take the risk of using such technology [14]. Therefore, we propose the following hypotheses:

H_3a_: Insecurity negatively influences nurses’ PEOU of MEMR.

H_3b_: Insecurity negatively influences nurses’ PU of MEMR.

#### Effect of discomfort on PEOU and PU

Discomfort refers to the perceived lack of control over technology and a feeling of being overwhelmed by it [9]. People who are uncomfortable with technology believe that they are controlled by it and that technologies are not suitable for common people [9]. That is, they may tend to have anxious feelings about the use of technology, a similar construct to computer anxiety, which has been confirmed to have a negative impact on PEOU [72] and PU [73]. According to previous literature, the following hypotheses are proposed:

H_4a_: Discomfort negatively influences nurses’ PEOU of MEMR.

H_4b_: Discomfort negatively influences nurses’ PU of MEMR.

#### Effect between PEOU and PU

PEOU refers to the extent to which a person believes that using the system will be free of effort, and PU refers to the extent to which a person believes that using the system will enhance their job performance [21]. According to TAM [6], PU is also affected by PEOU. In other words, the easier the system is to use, the more useful it can be [21]. However, there are studies that do not support this relationship [23,24,51]. The relationship between PEOU and PU is still inconclusive and requires more in-depth investigation. Thus, we propose the following hypothesis:

H_5_: Nurses’ PEOU of MEMR positively influences their PU of MEMR.

#### Effect among PEOU, PU, and behavioral intention

TAM suggests that two specific behavioral beliefs, PEOU and PU, collectively decide an individual’s behavioral intention to use a technology [6]. The effects of PEOU and PU on behavioral intention to use technology have been validated by several studies in the healthcare context [23,24,35,39]. Nevertheless, there are also studies which do not support the relationships between PEOU or PU toward behavioral intention [39,51]. Thus, the results are also still inconclusive and more investigations may be required. Consequently, this study proposes the following hypotheses:

H_6_: Nurses’ PEOU of MEMR positively influences their behavioral intention to use MEMR.

H_7_: Nurses’ PU of MEMR positively influences their behavioral intention to use MEMR.

## Methods

### Measurement development

The study measured optimism, innovativeness, discomfort, and insecurity using nine, seven, eight, and nine items, respectively, which were taken from Parasuraman’s instrument [9], and these items were modified according to experts’ suggestions. The instruments for PEOU, PU, and behavioral intention utilized three, five, and three items, respectively, and were adapted from previous empirically validated studies [6,74].

This survey adopted a five-point Likert scale questionnaire (5 for strongly agree and 1 for strongly disagree) which is illustrated in Additional file 1. The questionnaire draft used in this study consisted of one cover page and questions. The cover page briefly introduced the purpose of the study and practically defined EMR and MEMR. The draft was then reviewed by experts, including two healthcare information management researchers and two nursing management practitioners. Then, an in-depth interview and a pretest were conducted on five experienced nurses, whose opinions were compiled as the modification reference for the final version of the questionnaire. As a result, the final questionnaire should possess sufficient content validity and face validity [75]. The original questionnaire had 44 questions designed to cover the seven variables.

### Data collection and ethics approval

At the request of the hospital nursing department of a 1300-bed Taiwanese hospital employing about 900 nurses, this study used a cross-sectional design with data collected from the nurses of the hospital. The study chose the hospital for two primary reasons: (1) the subject hospital provides nearly all necessary medical services, which attracts an average of nearly 5,000 outpatients each day; and (2) the subject hospital is equipped with a well-established Hospital Information Systems and EMRs systems that provide patients with high quality services. Further, the subject hospital has introduced mobile EMRs system since 2010, which also makes the hospital suitable for studying MEMR. Regarding MEMR, its main function is to help nurses complete their nursing records at the bedside to enhance point of care for patients. Further, nurses can also access and manage EMRs anywhere and anytime. In order to protect the rights and privacy of the participants, appropriate ethical approval for this study was obtained from the Institutional Review Board of the hospital (Chi-Mei Medical Center, Taiwan) before the questionnaires were officially distributed to all of the nursing stations.

## Results

A total of 878 questionnaires were distributed to all of the 878 full-time registered nurses in wards during December 2010 and, finally, 665 useful ones were collected yielding a response rate of 75.74%. As summarized in Table 2, most respondents were female (658 cases, 98.95%); regarding unit type, the largest response was from the intensive care unit (197 cases, 29.62%); regarding nursing professional level, the largest sector was from N2 (210 cases, 31.58%); regarding age, the largest sector was 26–30 years old (329 cases, 49.47%).

**Table 2:** Descriptive statistics of respondents

**Background**	**Category**	**# of responses (%)**
Gender	Female	658(98.95%)
	Male	7(1.05%)
Unit type	Internal medicine series	192(28.87%)
	Surgery series	180(27.07)
	Gynecology and pediatrics series	79(11.88%)
	Intensive care unit series	197(29.62%)
	Missing	17(2.57%)
Nursing professional level	N0	77(11.58%)
	N1	101(15.19%)
	N2	210(31.58%)
	N3	114(17.14%)
	N4	29(4.36%)
	Team leader	98(14.74%)
	Head nurse	36(5.42%)
Age	Under 25	130(19.55%)
	26-30	329(49.47%)
	31-35	152(22.86%)
	36-40	40(6.02%)
	41-45	9(1.35%)
	Over 45	3(0.45%)
	Missing	2(0.30%)

The present study uses Partial Least Square (PLS), a component-based structural equation modeling [76], supported by SmartPLS® 2.0 M3 software for the analysis. Previous studies [76] suggested a two-step process for the assessment of the PLS model structure, encompassing (1) the assessment of the measurement model and (2) the assessment of the structural model. The study used PLS to assess the factorial validity of the measurement items. Based on the item loadings belonging to their respective constructs, a total of the loadings of 15 items (one item of optimism, one item of innovativeness, six items of insecurity, and seven items of discomfort) was lower than the cut-off value of 0.7 [77]. After removing these 15 items, a second-run factor analysis was performed and the results indicated that all factor loadings were higher than 0.7.

The criteria for the assessment of reliability often include Cronbach’s α and composite reliability (CR) [76]. The Cronbach’s α of all the variables were higher than the suggested value of 0.7 [76], while the CR of all the variables were greater than 0.8, which were higher than the suggested cut-off value of 0.7 [76] (Table 3) indicating sufficient reliability of the measurement.

**Table 3:** Reliability analysis

**Dimensions**	**CR**	**Cronbach’s α**	**Mean**	**Std. dev.**
Optimism	0.96	0.95	4.02	0.62
Innovativeness	0.93	0.91	3.44	0.69
Insecurity	0.87	0.77	2.01	0.59
Discomfort	0.87	0.78	3.22	0.75
Perceived ease of use	0.95	0.93	3.95	0.76
Perceived usefulness	0.94	0.93	3.94	0.78
Behavioral intention	0.92	0.87	4.32	0.62

The criteria for assessing validity include convergent validity and discriminant validity [76]. Fornell and Larcker [77] suggest using the average variance extracted (AVE) as a criterion of convergent validity. The results revealed that AVE ranged between 0.68 and 0.87, exceeding the cut-off value of 0.5 [77], suggesting good convergent validity (See Table 3). Moreover, discriminant validity can be assessed by comparing the square root of the AVE and the correlations of the construct with the other constructs in the model [77]. The results indicated that none of the intercorrelations of the constructs employed in the study exceeded the square root of the AVE for the construct (See Table 4), indicating satisfactory discriminant validity.

**Table 4:** Correlation matrix

	**AVE**	**A**	**B**	**C**	**D**	**E**	**F**	**G**
Optimism (A)	0.73	**0.85**						
Innovativeness (B)	0.68	0.54	**0.82**					
Insecurity (C)	0.69	−0.39	−0.26	**0.83**				
Discomfort (D)	0.69	−0.08	0.03	−0.12	**0.83**			
Perceived ease of use (E)	0.87	0.54	0.36	−0.29	−0.14	**0.93**		
Perceived usefulness (F)	0.77	0.54	0.32	−0.24	−0.12	0.67	**0.88**	
Behavioral intention (G)	0.79	0.54	0.27	−0.39	−0.11	0.56	0.52	**0.89**

Note: The bold numbers on the leading diagonal show the square root of the variance shared by the constructs and their measures.

Since the study collected both independent and dependent variables simultaneously from the same respondent, common method bias might be a concern [78]. The Harman’s single factor test [79] was used to ensure that there was no significant method effect on the causal relationships. The exploratory factor analysis reveals that more than two factors can be extracted, the first factor explaining about 29% of variance. Furthermore, the study randomized the items when surveying the samples, which may also reduce common method bias [75]. Thus, the common method bias should not be an issue in this study.

**Figure 2:**
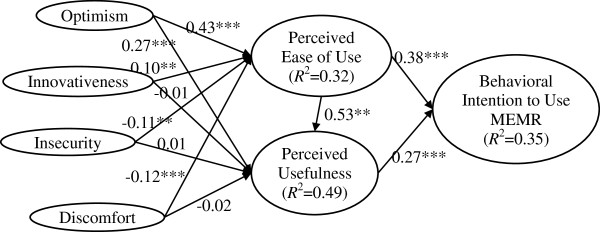
Structural model results.

The estimates for path coefficients focus on the sign, magnitude, and significance of the path relationships in the structural model [76]. Regarding the significance testing of the path coefficients, the study employed the bootstrapping resampling strategy. Based on the results, eight out of eleven hypotheses (H_1a_, H_1b_, H_2a_, H_3a_, H_4a_, H_5_, H_6_, and H_7_) were supported. Optimism, innovativeness, discomfort, and insecurity jointly explained about 32.06% of PEOU, while PU was explained by about 49.29% of variance. Further, the model explained about 35.16% of behavioral intention’s variance. These figures are lower than average TAM studies. However, they are higher than other healthcare studies that also recruited nurses as subjects [29,31,32]. As far as the authors are aware of, only Wu et al. [44] and Kummer et al. [80] achieved higher results. Properties of the causal path, including standardized path coefficients and hypotheses testing results, are presented in Table 5 and Figure 2.

**Table 5:** Summary of hypothesis testing results

**Hypothesis**	***p*-value**	**Results**
H_1a_: Optimism→Perceived ease of use	<0.001***	Supported
H_1b_: Optimism→Perceived usefulness	<0.001***	Supported
H_2a_: Innovativeness→Perceived ease of use	0.007**	Supported
H_2b_: Innovativeness→Perceived usefulness	0.636	Failed to support
H_3a_: Insecurity→Perceived ease of use	0.007**	Supported
H_3b_: Insecurity→Perceived usefulness	0.744	Failed to support
H_4a_: Discomfort→Perceived ease of use	<0.001***	Supported
H_4b_: Discomfort→Perceived usefulness	0.511	Failed to support
H_5_: Perceived ease of use→Perceived usefulness	<0.001***	Supported
H_6_: Perceived ease of use→Behavioral intention	<0.001***	Supported
H_7_: Perceived usefulness→Behavioral intention	<0.001***	Supported

Note: ** *p*<0.01, *** *p*<0.001.

Table 6 summarizes which relationships specified by the original TAM were significant and non-significant across studies conducted in healthcare context. Although the results of some studies are not in line with TAM, especially in healthcare settings, most studies, including this study, are in accordance with the results of TAM. Further discussions on the comparisons between our study and these TAM studies are presented in discussion section.

**Table 6:** Comparison of results of relationships specified by TAM studies in healthcare

**Relationship**	**Supported studies**	**Un-supported studies**	**% of supported studies**	**Results of this study**
PEOU→PU	[25-29,31-37,39,40,43-45,47-50]	[23,24,51]	21/24	Supported
PEOU→BI	[22,30,32,33,38,40,41,43],[44,48-50]	[24,27,28,34,36,37,39,46]	12/20	Supported
PU→BI	[24,27-30,32,33,36,37,39-41,43],[44,46-51]	[38]	20/21	Supported

Note: PEOU denotes perceived ease of use, PU denotes perceived usefulness, BI denotes behavioral intention.

## Discussion

### Effect of optimism on PEOU and PU

The research results showed that optimism has a significant effect on PEOU and PU. That is, the more optimistic nurses are, the more that PEOU and PU toward MEMR are perceived by nurses. The results are in agreement with previous research [8]. Optimism concerns the possession of positive attitude toward technology such as people’s beliefs of level of control, flexibility, convenience and efficiency [9], while it is important that people can assure that the technologies are under their control [81].

In nursing, technological optimists advocate the use of technology is an opportunity to broaden their professional field. If technology can be readily assimilated into humanistic nursing practice, nursing will be socially advantaged by technology [82]. It implies that the optimistic nurses are usually prone to accept the technologies for nursing care and believe that technology can make their work more efficient. That is, optimistic nurses will expect the MEMR can help accomplishing care work and having more confidence on the use the MEMR.

### Effect of innovativeness on PEOU and PU

The results of this study indicated that innovativeness has a significant effect on PEOU but not on PU. In other words, the more innovative nurses are, only more PEOU toward MEMR is perceived by nurses. The results of the relationship between innovativeness and PEOU are consistent with previous research [83]. People who are early adopters of innovative technology will use the technology even when the potential benefits are still inexplicit [8]. Previous study [65] also confirmed that the higher an individual’s personal innovativeness, the more effect on PEOU. Nowadays most people, including nurses, are familiar with new technologies such as smart phones, tablet pc, and so forth. These high innovative people may be more concerned about the "innovativeness" rather than the “practicality” about a new technology. They will be eager to try these new technologies and inclined to understand the new features and usage skills. The study findings also reveal that innovativeness was associated with PEOU.

In terms of the insignificant relationship between innovativeness and PU, the functions MEMR provided in the study site are mostly the same as traditional EMR systems except for the mobility and wireless features. Consequently, innovative nurses may not believe that MEMR will provide additional attractive or useful functions. This may explain why innovativeness does not influence PU.

### Effect of insecurity on PEOU and PU

The results of this study indicated that insecurity has a negative impact on PEOU. This finding was expected. The relationship between insecurity and PEOU is in agreement with previous literature [8]. That is, the more insecurity that nurses possess, the less PEOU towards MEMR is perceived by nurses. Nurses with the sense of technological insecurity may worry about the negative consequences of mobility and ubiquity such as complexity or their lack of trust in the system when using MEMR. For example, if unskillful nurses are required to use touch screen, smaller keyboard, and mouse simultaneously, they may create erroneous records due to the complexity of MEMR manipulation. Further, they may also concern whether the wireless network will cause patient privacy leakage or incomplete data storage. Therefore, the more insecurity characteristic nurses possess, they will worry more about being incapable of using MEMR gracefully. The results of this study may also reflect this phenomenon.

On the other hand, insecurity appeared to be unrelated to PU. Such a result is consistent with previous literature [45]. Nurses of technological insecurity may be more concerned about the lack of security protection for MEMR. As a result, whether the MEMR is useful for their nursing care may not be the first priority. After all, there exist not much difference between the functionalities MEMR and traditional EMR systems in the study site.

### Effect of discomfort on PEOU and PU

The results of this study revealed that discomfort negatively influenced PEOU. The results are consistent with previous literature [7,38]. This implies that if nurses perceive MEMR as discomfort, they will be more likely to perceive MEMR as being not easy to use. Uncomfortable nurses may have been accustomed to using existing or easier technologies; they may subconsciously reject to use high-tech or new technologies. Currently, the study site has adopted traditional desktop systems for decades and thus nurses are familiar with the usage style of existing EMR systems. The study site only implemented MEMR less than two years, thus, nurses may encounter the huge differences between “mobile vs. fixed” and “wireless vs. wired”. Uncomfortable nurses may be unable to adapt to the dramatic changes and worry about the use of MEMR, and thus subjectively regard MEMR as requiring lots of efforts.

On the other hand, discomfort appeared to be unrelated to PU. Such results are in agreement with previous study [54]. Uncomfortable nurses may feel unfamiliar with wireless and mobile operations of MEMR and thus cannot use MEMR comfortably. Consequently, whether MEMR is useful is not their top prioritized focus, this may explain why the construct of discomfort had no significant impact on PU.

### Effect of PEOU on PU and their effects on MEMR acceptance

In the past decade, many studies that used TAM have explained the important effect that PEOU has on behavioral intention to use an information system [20]. Consistent with the viewpoint in TAM, this study confirmed that PEOU has a positive effect on PU, while PEOU and PU both have direct effects on behavior intention. The relationships are also confirmed by healthcare studies regarding TAM [28,30,32,33,41,43,48-50,61]. However, the relationship between PEOU and PU is not supported in some healthcare studies [23,24,51], a possible explanation might be that specific medical technologies are often highly complex and the respondents did not have hands-on experience with them. Thus, the ease of use does not affect the intention to use a system if the usefulness is given. Also the ease of use does not influence the usefulness. However, nurses in this study are already familiar with the EMRs system since the system has been implemented for near two years. The primary difference is that EMRs system is now implemented on mobile devices. Thus, experience with MEMR should not be an issue for most nurses in this study. However, it needs further evidence to support this viewpoint. Further, the relationship between PU and behavioral intention in Schaper and Pervan’s study [38] is not supported, they argued that the possible reason is due to how the occupational therapists define job performance may be different.

Regarding the relationship between PEOU and behavioral intention, it may be the most controversial in healthcare studies that adopted TAM [20]. Many studies [24,27,28,34,36,37,39,46] reported insignificant results. Several studies [24,28,34] argued that the possible reason for the insignificant result is due to that more specialized subject population (e.g., physicians, healthcare professionals, etc.) may place a higher value on PU than on PEOU of a new technology. Consequently, they are willing to use the new technology even if it is complex. For nurses in this study, MEMR’s value is to make the fulfillment of their caring tasks easier and faster. Thus MEMR’s user-friendly appears indispensible for its usefulness.

In clinical practice, nurses always spend most of their time on face-to-face patient care activities such as medication administration, nursing assessment as well as a variety of patient treatments. They even resist information systems because they have to spend extra time on lengthy and trivial computer recording jobs during busy caring period [84]. Thus, designing easy to use MEMR is critical for nurses. If nurses perceive MEMR as easy to use, it will be easier for them to become familiar with the contents and functions MEMR provided and thus understand that MEMR is useful for them. That is, the extent of ease of use will influence nurses’ perception of usefulness regarding MEMR. Therefore, a user friendly interface for MEMR and the connectivity of networks are both important aspects when developing MEMR. Moreover, if MEMR is easy to use and useful to nurses’ care work, nurses will surely have higher intentions to use MEMR.

### A comparison of the results with previous studies

Compared with Walczuch and colleague’s study [8], there are two relationships that are not congruent: (1) the relationship between Innovativeness and PU, (2) the relationship between Insecurity and PEOU. Regarding the first inconsistent relationship, the possible reason might be that the technologies studied are different (see Table 7). This study investigated the same EMRs system for all nurses while Walczuch and colleague [8] investigated different software applications for each respondent. This means that respondents in Walczuch and colleague’s study [8] might perceive differently about how innovative the software applications are. On the other hand, the EMRs system is already implemented in the subject hospital, nurses are familiar with the functions of the system. Consequently, nurses in the subject hospital may perceive that EMRs on mobile is not innovative enough and causing the insignificant result in this study. Second, medical data is generally considered as more sensitive [85] and easily being leaked out [86], thus nurses may prioritize the issue of security over usefulness about MEMR. On the contrary, financial industries are highly regulated and less hacking events are reported [87,88]. Consequently, financial employees may value the usefulness of more than the security of software applications in Walczuch and colleague’s study [8] and caused the inconsistent results.

**Table 7:** Results of this study v.s. Walczuch et al.’s study

**Relationship**	**This study**	**Walczuch et al.’s study**
Optimism→PEOU	Support(+)	Support(+)
Optimism→PU	Support(+)	Support(+)
Innovativeness→PEOU	Support(+)	Support(+)
Innovativeness →PU	Non-support	Support(−)
Insecurity→PEOU	Support(−)	Support(−)
Insecurity→PU	Non-support	Support(−)
Discomfort→PEOU	Support(−)	Support(−)
Discomfort→PU	Non-support	Non-support
PEOU→PU	Support(+)	Support(+)

Note: (+) indicates positive relationship, (−) indicates negative relationship.

## Conclusions

### Summary of findings and contributions

This study conducted an empirical investigation in a large Taiwanese hospital to figure out the status of nurses’ technology readiness and to explore whether the four personality traits of TR, including optimism, innovativeness, discomfort, and insecurity, are associated with nurses’ perceptions of usefulness and ease of use of MEMR, and eventually influence nurses’ intentions toward MEMR use. Supported hypotheses were found for positive effects of optimism and innovativeness on nurses’ perceived ease of use of MEMR and negative effects of discomfort and insecurity on it. However, only optimism is associated with nurses’ perceived usefulness of MEMR. Furthermore, the causal relationships of perceived ease of use, perceived usefulness and behavior intention in TAM [6] were confirmed in this study as well.

One major limitation of TAM lies in that it does not help understand and explain acceptance in ways in addition to the perception of Usefulness and Ease of Use [7]. This may impede our ability to design meaningful interventions to promote HITs acceptance. Thus, the proposed model in this study considers personality traits, an important influencing factor but usually ignored in previous TAM research [8], as antecedents of PU and PEOU to better explain user acceptance of technology. By identifying nurses’ important personal traits that will influence their behavior intentions for using MEMR, the present study adds to the scientific literature by obtaining better understanding of technology acceptance. In addition, by adding personality traits to testing TAM in healthcare settings can not only help better understanding their impact on users’ behavioral intentions or actual use of MEMRs but also having benefits in developing general HIT theories. Further, TR is considered the individual’s “technological personality trait” and there have been numerous studies from different perspectives that explore the content and composition of TRI [9,14,69]. To the best of our knowledge, the impact of individual TRI on the acceptance of technology is still scarce [16,17], especially in the healthcare context [89]. The study empirically validated that personal TRI influences nurses’ acceptance of MEMR via PEOU and PU. Therefore, this study can help diminishing the gap in the influence of personality traits on technology acceptance.

### Implications and suggestions

Several implications for researchers and hospitals can be drawn from the results of this study. From a theoretical point of view, this study has contributed to the knowledge of applying TAM to predict nursing staff’s intention of new technology adoption. As expected, PU and PEOU both expressed the significance while explaining nurses’ behavior intentions toward MEMR use. Moreover, this study extended previous studies of the acceptance of mobile technologies and providing a greater insight into MEMR adoption at the basis of personal traits of technology readiness. This study had validated the TRI’s measurement scale within healthcare context also enrich the generalization of TRI applications.

As for managerial implications, nurses in this study demonstrating higher technological personalities of optimism, innovativeness and security will encourage hospitals to provide them with only appropriate technology assistance. However, the moderate low comfort (i.e. higher discomfort) of technology also implies that hospitals should provide nurses more compatible and simple technologies to mitigate their worry and resistance about the technology use. In other words, nurses have been ready to accept innovative technology with optimism and are less concerned with security. Further, among healthcare professionals, nurses are usually deficient in computer literacy [90] and thus may experience more computer anxiety and negative attitudes/expectations than the others. Thus, continuous education and training programs can be provided for nurses to enhance their information technology literacy, help them realizing the benefits of information technology, and minimize their stress and discomfort about information technology. In particular, hospitals should inform nurses that adopting technologies will not change their work habits dramatically.

The study found that only optimism affected the PU of MEMR. It is, therefore, suggested that hospital should recruit, either internally or externally, more optimistic nurses as champions of MEMR by leveraging the TRI instrument proposed in this study. These nurses may then recommend MEMR to their colleagues after they fully comprehend the benefits of MEMR. Moreover, PEOU and PU are factors that affect the nurses' willingness to use MEMR. Therefore, it is also suggested that nurses’ requirements must be fully understood during the development of MEMR to ensure that MEMR can meet the real needs of nurses. Moreover, it is also important to focus on the friendliness of user interfaces of MEMR and the compatibility of nurses’ work practices as these will greatly enhance nurses’ willingness to use MEMR. Since the results of the present study demonstrated that personality traits influenced nurses’ acceptance of MEMR, thus the effects of TR should not be ignored, indicating that hospitals should also include more employees’ characteristics beyond socio-demographic profiles in their personnel databases.

### Limitations and future research

Combining TRI with TAM is the first step to obtain superior understanding of technology acceptance among differing individuals with different traits. Although most of the findings were significant, certain limitations existing in the study must be recognized. First, the sample of this study was collected from just one hospital in one country, which may reduce the results’ generalizability to other organizations and countries. In addition, the representativeness of the respondents may also be limited. Second, the study was conducted in a cross-sectional perspective, which may lead to a snapshot presentation of the current study. Thus, it would add more value to the proposed model if longitudinal studies are carried out. Third, while the current study was focused on nurses’ personality traits on the acceptance of MEMR, future studies could investigate different staff members, such as physicians, in order to gain a better knowledge of the influence of personal dispositions. Future research could also investigate which new constructs, such as perceived trust and perceived risk, could add to the explained variance of the proposed model. Fourth, the study could be completed in different settings to compare the results. A qualitative study regarding the discomfort of nurses could be another possibility for further research. Fifth, this study did not investigate actual use of MEMR which might be unable to show the explanation is valid for the behavior of interest. Sixth, experience was not considered as an influence factor in this study because all nurses shared at least some experience with related systems. However, the degree of experience could differ individually and may influence the results. Finally, gender bias due to the sample (98.95% females) should also be noticed when citing the results.

## Competing interests

The authors declare that they have no competing interests.

## Authors’ contribution

KMK and CFL conceived of this study and participated in the its design and carried out the study. KM, CF, and CCM drafted the manuscript and performed the statistical analysis. All authors read and approved the final manuscript.

## Pre-publication history

The pre-publication history for this paper can be accessed here:

http://www.biomedcentral.com/1472-6947/13/88/prepub

## Supplementary Material

Additional file 1 Questionnaire items. Click here for file
