# Supplementary material for: An investigation of the effect of nurses’ technology readiness on the acceptance of mobile electronic medical record systems
Source: BMC Med Inform Decis Mak. 2013 Aug 12;13:88. doi: 10.1186/1472-6947-13-88 (PMC3750758; doi:10.1186/1472-6947-13-88)
Supplement: Additional file 1 — Questionnaire items. [file 1472-6947-13-88-S1.doc]

**Additional file 1. Questionnaire items**

| Variable | Item |
| --- | --- |
| Optimism [9] | Technology gives people more control over their daily lives |
|  | Products and services that use the newest technologies are much more convenient to use |
|  | You like the idea of doing business via computers because you are not limited to regular business hours |
|  | You prefer to use the most advanced technology available |
|  | You like computer programs that allow you to tailor things to fit your own needs |
|  | Technology makes you more efficient in your occupation |
|  | Technology gives you more freedom of mobility |
|  | Learning about technology can be as rewarding as the technology itself |
|  | You feel confident that machines will follow through with what you instructed them to do |
| Innovativeness [9] | Other people come to you for advice on new technologies |
|  | It seems your friends are learning more about the newest technologies than you are |
|  | In general, you are among the first in your circle of friends to acquire new technology when it appears |
|  | You can usually figure out new high-tech products and services without help from others |
|  | You keep up with the latest technological developments in your areas of interest |
|  | You enjoy the challenge of figuring out high-tech gadgets |
|  | You find you have fewer problems than other people in making technology work for you |
| Discomfort [9] | Technical support lines are not helpful because they do not explain things in terms you understand |
|  | Sometimes, you think that technology systems are not designed for use by ordinary people |
|  | There is no such thing as a manual for a high-tech product or service that is written in plain language |
|  | When you get technical support from a provider of a high-tech product or service, you sometimes feel as if you are being taken advantage of by someone who knows more than you do |
|  | If you buy a high-tech product or service, you prefer to have the basic model over one with a lot of extra features |
|  | It is embarrassing when you have trouble with a high-tech gadget while people are watching |
|  | There should be caution in replacing important people-tasks with technology because new technology can breakdown or get disconnected |
|  | Many new technologies have health or safety risks that are not discovered until after people have used them |
| Insecurity [9] | You do not consider it safe giving out a credit card number over a computer |
|  | You do not consider it safe to do any kind of financial business online |
|  | You worry that information you send over the Internet will be seen by other people |
|  | You do not feel confident doing business with a place that can only be reached online |
|  | Any business transaction you do electronically should be confirmed later with something in writing |
|  | Whenever something gets automated, you need to check carefully that the machine or computer is not making mistakes |
|  | The human touch is very important when doing business with a company |
|  | When you call a business, you prefer to talk to a person rather than a machine |
|  | If you provide information to a machine or over the Internet, you can never be sure it really gets to right place |
| Perceived Ease of Use [21] | Your interaction with MEMR is clear and understandable |
|  | It is easy for you to remember how to perform tasks using MEMR |
|  | Overall, you believe that MEMR is easy to use |
| Perceived Usefulness [21] | Using the MEMR in your job increases your productivity (e.g., medication recording) |
|  | Using the MEMR enhances your job quality (e.g., increasing medicating immediacy, decreasing medicating error) |
|  | You find MEMR to be useful in your job |
|  | Using the MEMR improves your performance in your job |
|  | Using the MEMR enhances your effectiveness in your job |
| Behavioral Intention [74] | You will use MEMR on a regular basis in the future |
|  | You will frequently use MEMR in the future |
|  | You will strongly recommend other nurses to use MEMR |
